# Localized Acquired Hypertrichosis Associated with the Application of a Splint

**DOI:** 10.1155/2012/592092

**Published:** 2012-03-26

**Authors:** Alexander K. C. Leung, Andrew S. Wong

**Affiliations:** ^1^Department of Pediatrics, University of Calgary, Alberta Children's Hospital, AB, Canada T2M 0H5; ^2^Department of Family Medicine, University of Calgary, Calgary, AB, Canada T2M 0H5

## Abstract

We describe a 16-year-old boy whose left forearm and hand were cut by a piece of glass from a broken window as a result of the fall. He had surgical repair of his left extensor pollicis brevis, abductor pollicis brevis, and dorsal branch of the left radial nerve. Following the surgery, he was put on a splint so as to immobilize the left forearm and wrist. On removal of the splint 4 weeks post surgery, he was noticed to have more hair growth on his left forearm and hand than his right counterparts. The patient was reassessed 2, 4, and 8 months after the removal of the splint. The hypertrichosis got better with time. At the last visit, the hair growth in the left forearm and hand was back to normal. Our patient represents the first reported case of localized acquired hypertrichosis following the application of a splint in the pediatric literature.

## 1. Introduction

Hypertrichosis refers to the increased growth of vellus or other hair at inappropriate locations beyond the normal variation for a patient's reference group [1]. The affected areas have a greater number of hair follicles than is normal for the body site [1, 2]. The condition is unrelated to androgen excess and unaccompanied by virilism or menstrual abnormalities. The sudden appearance of excessive hair growth in a localized body area following application of a splint has rarely been described. A perusal of the literature reveals only two documented cases. We describe a 16-year-old boy who developed hypertrichosis in the left forearm and hand following the application of a splint. To our knowledge, our patient represents the first patient who developed hypertrichosis following the application of a splint in the pediatric age group.

## 2. Case Report

 A healthy 16-year-old Chinese boy fell downstairs 6 weeks ago. His left forearm and hand were cut by a piece of glass from a broken window as a result of the fall. He was taken to a local hospital where he had surgical repair of his left extensor pollicis brevis, abductor pollicis brevis, and dorsal branch of the left radial nerve. Following the surgery, he was put on a splint so as to immobilize the left forearm and wrist. On removal of the splint 4 weeks after surgery, he was noticed to have more hair growth on his left forearm and hand than his right counterparts.

**Figure 1:**
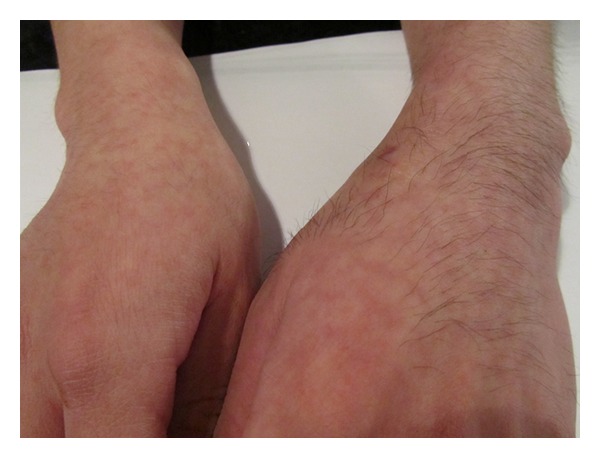
Hypertrichosis of the left forearm and hand following the application of a splint.

 On physical examination, there was increased hair growth in the left forearm and hand over the area in which the splint was located (Figure 1). There was a surgical scar on the left forearm, wrist, and hand. The left forearm and thenar area of the left hand had less musculature than the right counterparts. Range of motion was normal in the wrists, elbows, and hands. Wrist and thumb strength on the left side was slightly less than the right side (4+ versus 5). The rest of the neurological examination was normal.

 The patient was reassessed 2, 4, and 8 months later. The hypertrichosis got better with time. At the last visit, the hair growth in the left forearm and hand was back to normal.

## 3. Discussion

Hypertrichosis can be generalized or localized and may be congenital or acquired. Congenital generalized hypertrichosis may result from maternal ingestion of medications (such as minoxidil, phenytoin, and diazoxide) or alcohol, or it may be inherited in an autosomal dominant pattern (e.g., hypertrichosis lanuginosa, universal hypertrichosis, or hypertrichosis with gingival hyperplasia) or an X-linked dominant pattern [1]. Congenital localized hypertrichosis is a notable feature of congenital melanocytic nevi, congenital Becker nevi, nevoid hypertrichosis, nevus pilosus, smooth muscle hamartomas, plexiform neurofibromas, and linear epidermal nevi [1]. Acquired generalized hypertrichosis is most frequently caused by medications, such as phenytoin, cyclosporine, danazol, minoxidil, penicillamine, diazoxide, psoralens, anabolic agents, corticosteroids, acetazolamide, hexachlorobenzene, and streptomycin [1, 2].

Acquired localized hypertrichosis is known to arise following chronic irritation, friction, or inflammation and may develop around chickenpox scars, the sites of insect bites, at the periphery of burned skin, and on the legs after radical inguinal lymphadenectomy [1–3]. The condition has also been noted after topical use of hydrocortisone, or fiberglass cast, after X-ray or UV irradiation, and in patients with mental illness who repeatedly bite or scratch their hands and arms [1, 3].

Hypertrichosis has also been reported following the application of a plaster of Paris or fibreglass cast [3–5]. In 1980, Pick described a 12-year-old boy who had an effusion of the right knee following a fall [4]. A plaster of Paris was applied to the knee area. When the cast was removed 6 weeks later, a prominent focal patch of hair was noted in the patella area. The hairy patch spontaneously resolved two months after the cast was removed. In 1983, Bergen reported a 31-year-old woman who had a Colles' fracture as a result of a fall on a cement floor [5]. She was treated with a circular plaster cast for 7 weeks. On removal of the cast, a localized patch of hair growth was noted where the plaster had been. In 1989, Leung et al. reported 3 children, aged 3.5, 5, and 7 years, respectively, who developed hypertrichosis following a fracture and cast application [3]. The 3.5-year-old boy had a fibreglass cast for 9 weeks. The 5-year-old girl had a plaster of Paris cast for 11 weeks, while the 7-year-old boy had serial Petrie cast treatment for 6 months. In 1985, Chang and Cohen reported a 29-year-old man who had localized hypertrichosis and dyshidrotic dermatitis on the left forearm following multiple fractures, which required application of a fibreglass cast for 2 months [6]. When the patient was reassessed 1 to 2 months later, the hypertrichosis had started to resolve. In 2001, Kara et al. reported a boy with pes equinovarus who had corrective surgery at 7 months of age [7]. At the age of 11 years, he presented to an orthopedic department because of leg pain and difficulty in walking. He had to be reoperated on and a calcaneal osteotomy was performed. After the operation, a cast was applied from the hip to the ankle of the right leg for 2 months. When the cast was removed, it was noted that there was mild atrophy and increased hair growth on the right leg as compared to the left. When he was seen 3 months later, the muscle wasting was no longer noted and there was complete, spontaneous resolution of the hypertrichosis.

Rarely, hypertrichosis has been reported following the application of a splint [8]. In 1986, Harper reported two young females, 22 and 23 years of age, respectively, who had closed fractures and developed hypertrichosis of the upper extremities after application of a splint for 6 to 12 weeks [8]. The hypertrichosis resolved over approximately 6 months. We reported a 16-year-old boy who developed hypertrichosis in the left forearm and hand following the application of a splint. To our knowledge, our patient represents the first case in the pediatric age group. It is conceivable that hypertrichosis following the application of a splint may be more common than is presently appreciated. With the report of this case, hopefully more case reports would be forthcoming.

It is postulated that hypertrichosis associated with application of plaster of Paris cast, fibreglass cast, or splint may result from chronic irritation, friction, inflammation, heat, or cutaneous hyperaemia [3]. The condition is usually transitory. In the patient reported by Bergen, the persistence of the hair growth might be due to insufficient followup as the patient was not reassessed after one month from the time the cast was removed. Physicians should be aware of the benign nature of this condition so that unnecessary investigations can be avoided.
